# An Analysis by the European Committee on Organ Transplantation of the Council of Europe Outlining the International Landscape of Donors and Recipients Sex in Solid Organ Transplantation

**DOI:** 10.3389/ti.2022.10322

**Published:** 2022-07-19

**Authors:** Emanuele Cozzi, Marina Álvarez, Mar Carmona, Beatriz Mahíllo, John Forsythe, Mar Lomero, Marta López-Fraga, Ruth Sapir-Pichhadze, Massimo Cardillo, Beatriz Domínguez-Gil

**Affiliations:** ^1^ Centro Nazionale Trapianti-Istituto Superiore di Sanità (CNT-ISS), Rome, Italy; ^2^ Department of Cardiac, Thoracic and Vascular Sciences, Transplant Immunology Unit, Padua University Hospital, University of Padua, Padua, Italy; ^3^ Organizacion Nacional de Trasplantes (ONT), Madrid, Spain; ^4^ NHS Blood and Transplant (NHSBT), Bristol, United Kingdom; ^5^ European Directorate for the Quality of Medicines & HealthCare (EDQM), Council of Europe, Strasbourg, France; ^6^ Division of Nephrology and Multi-Organ Transplant Program, Department of Medicine, McGill University, Montreal, QC, Canada

**Keywords:** donors, sex, inequalities, recipients, Council of Europe

## Abstract

Discrepancies in donation and transplantation by sex and gender have previously been reported. However, whether such differences are invariably the inevitable, unintended outcome of a legitimate process has yet to be determined. The European Committee on Organ Transplantation of the Council of Europe (CD-P-TO) is the committee that actively promotes the development of ethical, quality and safety standards in the field of transplantation in Europe. Whilst the ultimate objective is to shed light on the processes underlying potential gender inequities in transplantation, our initial goal was to represent the distribution by sex among organ donors and recipients in the CD-P-TO Member States and observer countries. Our survey confirms previous evidence that, in most countries, men represent the prevalent source of deceased donors (63.3% in 64 countries: 60.7% and 71.9% for donation after brain and circulatory death, respectively). In contrast, women represent the leading source of organs recovered from living kidney and liver donors (61.1% and 51.2% in 55 and 32 countries, respectively). Across countries, most recovered organs are transplanted into men (65% in 57 countries). These observations may be explained, at least in part, by the higher burden of certain diseases in men, childbearing related immune sensitization in women, and donor-recipient size mismatch. Future research should establish whether gender-related socially-constructed roles and socioeconomic status may play a detrimental role reducing the access of women to transplantation.

## Graphical Abstract

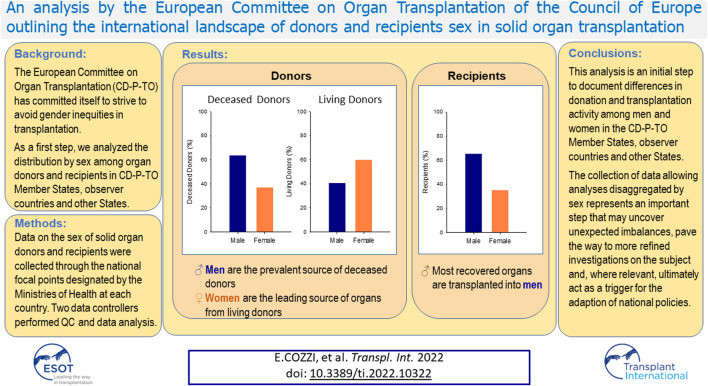

## Introduction

Sex and gender represent two fundamental variables that must. be taken into due consideration to ensure health policies are efficient and adapted to the current needs and circumstances of the global population (1). Accordingly, the European Committee on Organ Transplantation of the Council of Europe (CD-P-TO)^1^ has committed itself to take into account the impact of gender and sex in the performance of its tasks and to strive to avoid inequities in each of its policy areas.

To date, the terms gender and sex have often been used interchangeably. However, gender and sex have very specific meanings and must be applied in well-defined and distinct circumstances. Whilst sex exclusively refers to biological traits, gender regards non-biological attributes that are socially constructed and are the ultimate result of an individual’s roles, culture, and conventions (2–3).

Gender inequities in access to transplantation were previously reported (4–8). However, to the best of our knowledge, the sex of donors and recipients of solid organ transplants across the countries represented in the Council of Europe has not been investigated to date. Appreciating the importance of studying determinants of potential gender inequities in transplantation at the international level, the CD-P-TO decided, as an initial step, to collect data on the sex of solid organ donors and recipients in its annual data collection on donation and transplantation activities. These data are made available through “Newsletter Transplant”, the official annual publication of the Committee. Here we report the main findings of analyses conducted using the data provided for the year 2019 by Member States of the Council of Europe, Observer Countries, and other States. Indeed, the figures regarding the year 2019 represent the latest set of data that were not impacted by the Covid-19 pandemic.

## Methods

To investigate inequities in organ transplantation, questions on the sex of living and deceased organ donors and recipients were incorporated into the questionnaire that the Organización Nacional de Trasplantes (ONT) submits yearly to countries participating in the Newsletter Transplant (available at www.edqm.eu/freepub).

As far as deceased organ donors are considered, countries were first invited to provide national figures (absolute numbers). Subsequently, countries were asked to stratify the data by deceased donor type into donors after brain death (DBD) and donors after circulatory determination of death (DCDD). Countries were then requested to further provide the distribution of donors by sex. To examine the situation relating to living donation, countries were likewise invited to provide national figures relating to the sex of living kidney donors (LKD) and living liver donors (LLD). Finally, countries were also asked to provide data on the sex of recipients of solid organ transplants originating from both deceased and living donors.

The questionnaire was completed by national focal points designated by the Ministries of Health at each country. ONT then compiled the information collected by the questionnaires, performed the corresponding quality control of the data reported, and the analysis. Quality control of the data involved the review of each questionnaire by two data controllers. In the presence of inconsistencies, the ONT contacted the designated focal point in each country for a final data check. Analyses were carried out using SPSS v.25.0 and Excel. To calculate rates per million population (PMP), the country population was obtained from the United Nations Population Fund (UNFPA) report (www.unfpa.org).

## Results

### Participating Countries

A total of 69 countries responded to this initiative and provided thorough information on the sex of donors and recipients. In particular, the countries involved in the study include 36 Council of Europe Members States (Armenia, Austria, Belgium, Bulgaria, Croatia, Cyprus, Czech Republic, Denmark, Estonia, Finland, France, Germany, Greece, Hungary, Iceland, Ireland, Italy, Latvia, Lithuania, Luxembourg, Malta, Netherlands, Norway, Poland, Portugal, Republic of Moldova, Republic of North Macedonia, Romania, Russian Federation, Slovakia, Slovenia, Spain, Sweden, Switzerland, Turkey, United Kingdom), 3 Observer Countries (Mexico, Israel, and United States), 15 countries of Iberoamerican Network/Council of Donation and Transplantation- RCIDT (Argentina, Bolivia, Brazil, Chile, Colombia, Costa Rica, Cuba, Dominican Republic, Ecuador, Guatemala, Nicaragua, Panama, Paraguay, Peru, Uruguay, Venezuela) and 15 additional countries from 4 continents (Algeria, Australia, Belarus, China, India, Japan, Kuwait, Malaysia, Mongolia, New Zealand, Qatar, Saudi Arabia, Sudan, Syrian Arab Republic, United Arab Emirates).

### Sex of Deceased Organ Donors

**FIGURE 1:**
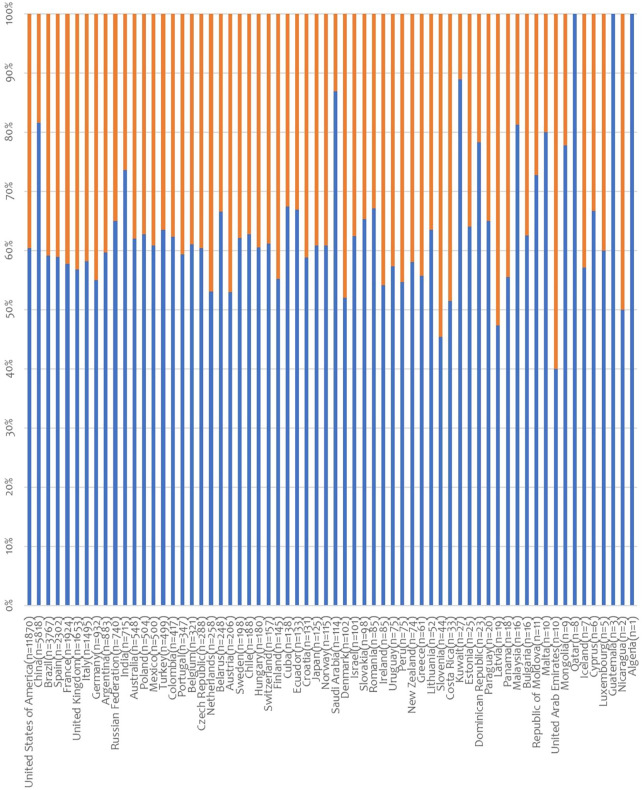
Distribution of deceased organ donors (DBD and DCDD) by sex. Data on donor sex was provided by 64 countries (in brackets: number of donors; blue lines: % male donors; orange lines: % female donors).

**FIGURE 2:**
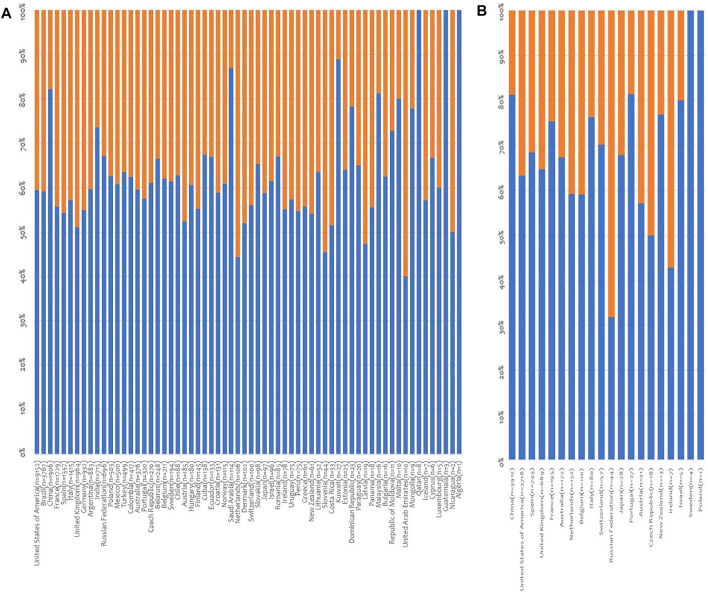
Distribution of deceased organ donors by sex and donor type. **(A)** Distribution of DBD by donor sex. Data on donor sex was provided by 64 countries; **(B)** Distribution of DCDD by donor sex. Data on donor sex was provided by 20 countries (in brackets: number of donors; blue lines: % male donors; orange lines: % female donors).

Globally, in 2019 there were 38,983 deceased organ donors recorded in the 69 participating countries. In the latter, DBD and DCDD activity was reported in 65 and 20 countries, respectively. Deceased donors PMP ranged from 0 to 49.6 (Supplementary Figure S1). Information about sex was available for 38,980 deceased donors (99.9%) in 64 countries, and men added up to 63.3% of these (Figure 1, Supplementary Table S1). When deceased donors were divided into DBD and DCDD donors, once again the percentage of male donors was prevalent (60.7% and 71.9% for DBD and DCDD, respectively). Except for 4 countries (United Arab Emirates, Slovenia, Latvia, and Nicaragua), the majority of deceased donors were invariably represented by men (range: from 40% to 100%). In all countries but 5 (United Arab Emirates, Slovenia, Latvia, Netherland, and Nicaragua), the percentage of female DBD never exceeded that of males (Figure 2A). Similarly, in all countries but 3 (Russian Federation, Ireland, and Czech Republic), the percentage of female DCDD never exceeded that of males (Figure 2B). Interestingly, in the case of deceased donors, an average of 2,67 organs could be retrieved from each donor.

### Sex of Living Donors

**FIGURE 3:**
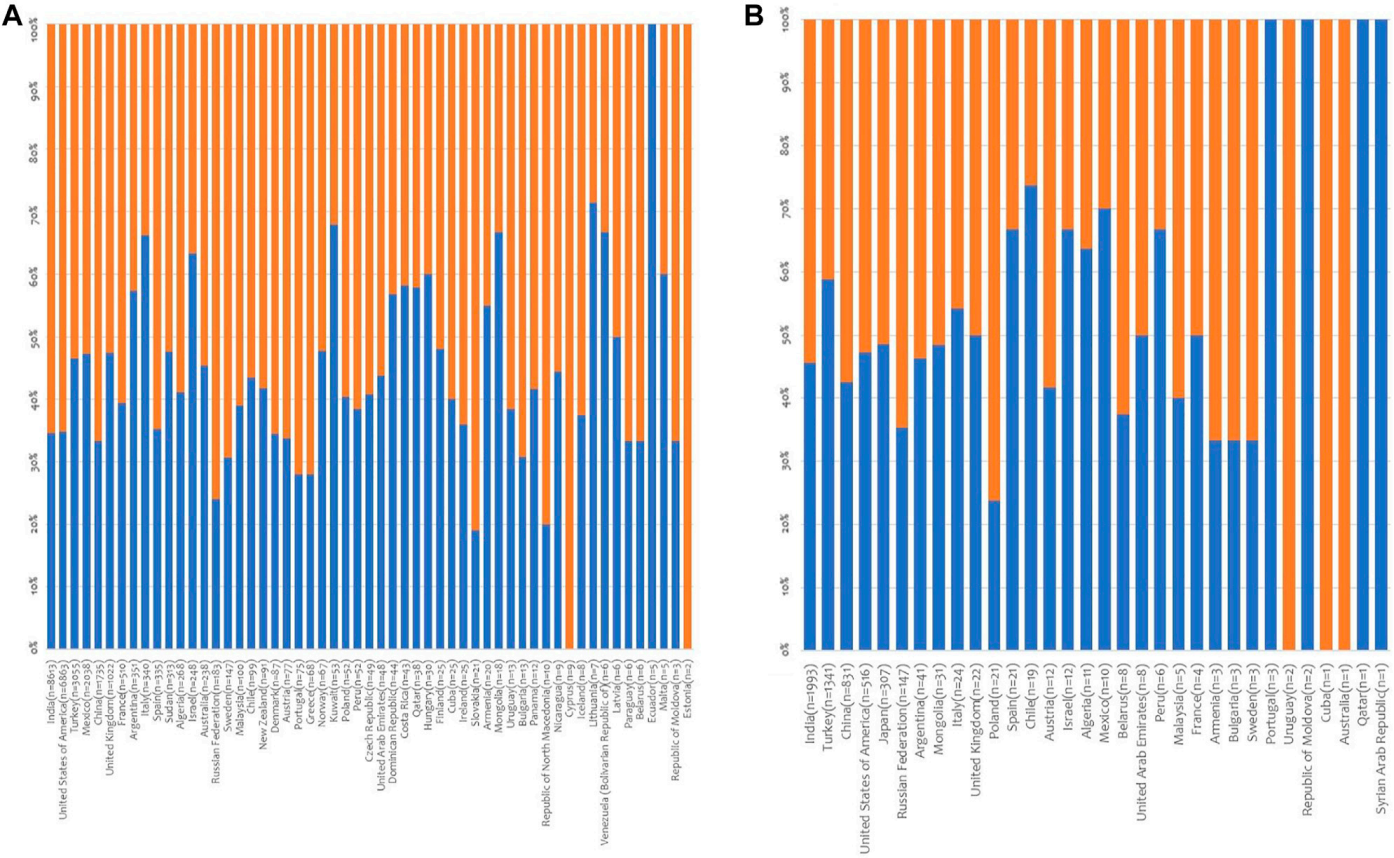
Distribution of living donors by sex. **(A)** Distribution of living kidney donors by sex. Data on donor sex was provided by 55 countries; **(B)** Distribution of living liver donors by sex. Data on donor sex was provided by 32 countries (in brackets: number of donors; blue lines: % male donors; orange lines: % female donors).

Internationally, there were 39,090 living donors (33,116 LKD and 5,974 LLD) recorded in the period considered. Information about sex was available for 32996 living donors (84.4%), and women added up to 59.5% of these. As far as LKD, a therapeutic approach that takes place in 67 of the participating countries, information about sex was available for 27586 donors (83.3%). Women accounted for 61.1% of the LKD ranging from 0 (Ecuador) to 100% (Estonia and Cyprus) (Figure 3A, Supplementary Table S2). Except for Ecuador, Lithuania, Kuwait, Venezuela, Mongolia, Italy, Israel, Malta, Hungary, Costa Rica, Qatar, Argentina, Dominican Republic, Armenia and Latvia, in reporting countries women accounted for the majority of LKD.

Similarly, as far as LLD, a therapeutic approach available in 40 of the participating countries, information about sex was available for 5,410 donors (90.6%). Female donors accounted for 51.2% of the livers transplanted, ranging from 0 (Portugal, Moldova, Syria and Qatar) to 100% (Uruguay, Australia and Cuba) (Figure 3B, Supplementary Table S2) Except for Portugal, Moldova, Syria, Qatar, Chile, Mexico, Peru, Israel, Spain, Algeria, Turkey, Italy, France, UAE and UK, in reporting countries women accounted for the majority of living liver donors.

Altogether, it is of interest that, in contrast to DD, for both kidney and liver the percentage of women amongst living donors exceeded that of men.

### Sex of the Patients Transplanted

**FIGURE 4:**
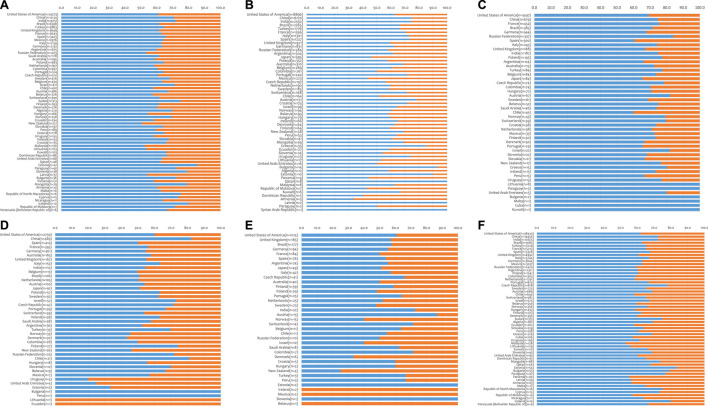
Distribution of solid organ transplant recipients by sex. **(A)** kidney transplant recipients (62 countries); **(B)** liver transplant recipients (56 countries); **(C)** heart transplant recipients (47 countries); **(D)** lung transplant recipients (42 countries); **(E)** pancreas transplant recipients (37 countries); **(F)** all transplant recipients (57 countries).

Finally, our studies have been extended to determine the sex of the recipients of the organs allocated in 2019 (N = 139,230) in the participating countries for which information about sex was available (N = 133,694, 96%), irrespective of the source of the organ implanted (deceased versus living donation) (Figure 4, Supplementary Table S3).

Men consistently received the vast majority of the organs transplanted in 2019 (65% of the total). In particular, men received 65% of the kidneys, 67% of the livers, 71% of the hearts, 60% of the lungs and 58% of the pancreases available. At a national level, men received the majority of the kidneys, livers, hearts, lungs and pancreases in 100%, 84%, 100%, 76%, and 68% of the surveyed countries, respectively.

## Discussion

Transplantation represents the ideal treatment option for patients with terminal organ failure. In the case of end-stage renal disease, transplantation is associated with improved quality of life and increased life expectancy compared to any other form of kidney replacement therapy (9–11). Likewise, transplantation represents the only form of treatment for terminal heart, lung, or liver failure. Unfortunately, due to the limited availability of organs, transplantation is precluded in many patients who could benefit from such a treatment (11). In this light, to avoid inequities it is fundamental that access to transplantation is carefully regulated and is the legitimate outcome of a fair and transparent process. Sex and gender differences in access to transplantation have been observed previously for kidney, liver and heart transplantation (4–8). However, it is yet to be established whether these differences are invariably the inevitable, fortuitous outcome of a legitimate process.

In an effort to shed some light on potential inequities in access to transplantation, we commenced by collecting and analysing data on donation and transplantation activity by organ donors’ and recipients’ sex in the CD-P-TO Member States, observer countries, and other States. As previously reported (12), our analysis of data collected prior to the COVID-19 pandemic in 69 countries and 6 continents, confirms that in most but not all countries, men are the prevalent source of both DBD and DCDD deceased donors. In this regard, it is of interest that, coherently, individuals who meet the biological criteria and who may eventually become deceased donors are more frequently men hospitalized in intensive care units as a consequence of severe and unrecoverable acute brain injury (due trauma or stroke) (13).

In contrast, our study clearly demonstrates that women are the leading source of kidneys recovered from living donors. In a context where donor voluntariness is an important determinant (14, 15), this observation may be explained by the more generous and altruistic nature of women in comparison to men (16–19). Yet, it is also important to recall that certain situational, group specific, or individual factors might reduce the degree of voluntariness. For example, as a consequence of their social role, women may perceive it as their maternal or spousal duty to become living donors and help their child or partner (20). Additionally, women may feel more pressured to donate and may be made to feel less autonomous because of societal and socioeconomic pressures (15) as men are often the prevailing source of family income (17, 21). Interestingly, a study involving men and women who served as living kidney donors, did not demonstrate differences in psychosocial profiles or greater vulnerability to family pressure between them (22). Future analyses should re-evaluate differences in living kidney donation among men and women as the contribution of women to family earnings increases. In the context of living liver donation, on the other hand, the number of organs provided by women only marginally exceeded the number of livers provided by men.

In all cases, the majority of the organs recovered are transplanted into men. Several reasons may account for such an observation that is valid collectively but also for each of the organs considered separately. First, certain diseases more frequently affect men resulting in a larger number of men being waitlisted for transplantation. For instance, chronic liver diseases are more frequently observed in men. Likewise, men more often develop kidney diseases (23) and, in most countries, men represent the larger proportion of patients on dialysis due to end-stage renal disease. Second, women are not infrequently penalized in accessing transplantation due to their immunological profile. In particular, women listed for a transplant may present greater immune sensitization (measured by pre-transplant panel reactive antibodies (PRA)) as a consequence of previous pregnancies (24). Third, women may not be selected for transplantation due to donor-recipient size mismatch (25). However, other gender related factors may also be at play. For example, the interplay between psychosocial and cultural pressures on women, and subtle differences in perception of women as transplant candidates, limit the full use of transplant treatment options for women (26). A recent North American study, for example, showed that, whilst in men only a BMI ≥40 kg/m^2^ was associated with lower likelihood of transplantation from any donor source, in the case of women, BMI ≥25 kg/m^2^ was associated with a lower access to transplantation from both deceased and living donors (27). In the case of paediatric candidates, in addition to physician attitudes, patient and caretaker motivation toward transplantation may also contribute to gender inequity in girls’ access to pre-emptive transplants (28). In certain countries, limited education and health literacy (29) as well as socioeconomic dependence may affect some women. Future studies should shed light on patient, health care provider, and system-related factors that may contribute to reduced access to transplantation among women compared to men. Similarly, currently available data prevents us from verifying whether, at least in some countries, gender-related issues or socioeconomic variables may play a detrimental role, possibly reducing the access of women to the transplant waiting lists and, ultimately, to transplantation.

We would like to acknowledge several limitations of the current study. Information on sex was not available for all donors and recipients involved in the transplantation activity of the year considered in all the participating countries. Furthermore, the data collection undertaken did not enable an analysis of the findings according to the four possible donor-recipient sex combinations (M-M; M-F; F-F; F-M). Additionally, we did not have access to additional pertinent donor and recipient variables, including age, socioeconomic status, the relationship between donor-recipient pairs, and national statistics on organ failure and waiting lists among men and women (e.g., cause for end-stage disease, waiting time, death whilst listed for transplantation). Because of the cross-sectional nature of this study, we cannot rule out that our observations on the sex of donors and recipients in organ transplantation may have differed in preceding years or may change further as a consequence of the ongoing covid-19 pandemic. Finally, while our findings preclude a thorough assessment of the processes underlying potential inequities in access to transplantation by patients’ sex and gender, they represent an initial step in documenting the current state of affairs on their distribution among transplant donors and recipients at an international level.

In summary, this brief report is an initial step to document differences in donation and transplantation activity among men and women in the CD-P-TO Member States, observer countries and other States (69 countries in 6 continents). We are convinced that the collection of data allowing analyses disaggregated by sex represents an important step that may uncover unexpected imbalances, pave the way to more refined investigations on the subject and, where relevant, ultimately act as a trigger for the adaption of national policies. Accordingly, the CD-P-TO has decided to invest further resources into this research topic in the years to come. A follow up and more detailed questionnaire is expected to be submitted to the Health Authorities of the Council of Europe Member States in the second trimester of the year 2022.

## Data Availability

The raw data supporting the conclusion of this article will be made available by the authors, without undue reservation.

## Footnotes

1 The CD-P-TO is the steering committee in charge of organ, tissue and cell donation and transplantation activities at the European Directorate for the Quality of Medicines and HealthCare of the Council of Europe. It actively promotes the non-commercialization of organ, tissue and cell donation, the fight against organ trafficking and the development of ethical, quality and safety standards in the field of organ, tissue and cell transplantation. Its activities include the collection of international data and monitoring of practices in Europe, the transfer of knowledge and expertise between organisations and experts through training and networking and the elaboration of reports, surveys, and recommendations. As of November 2021, the CD-P-TO was composed of 39 members (Albania, Austria, Belgium, Bosnia and Herzegovina, Bulgaria, Croatia, Cyprus, Czech Republic, Denmark, Estonia, Finland, France, Germany, Greece, Hungary, Iceland, Ireland, Italy, Lithuania, Latvia, Luxembourg, Malta, Montenegro, Netherlands, North Macedonia, Norway, Poland, Portugal, Romania, Serbia, Slovak Republic, Slovenia, Spain, Sweden, Switzerland, Republic of Moldova, Turkey, Ukraine, and United Kingdom) and 22 observers (Armenia, Belarus, Canada, Georgia, Israel, Russian Federation, United States, Council of Europe Committee on Bioethics, DTI Foundation, European Association of Tissue and Cell Banks, European Commission, European Eye Bank Association, European Society for Blood and Marrow Transplantation, European Society for Organ Transplantation, European Society of Human Reproduction and Embryology, Eurotransplant, Scandiatransplant, South-Europe Alliance for Transplants (SAT), The Transplantation Society, United Network for Organ Sharing (UNOS), World Health Organization (WHO), and World Marrow Donors Association).

## Supplementary Material

Supplementary Figure S1 Deceased donor rates per million population (PMP). Data from 69 participating countries (in brackets: absolute number).

Supplementary Table S1 Distribution of deceased organ donors (DBD and DCDD) by sex. Data on donor sex was provided by 64 countries.

Supplementary Table S2 Distribution of living kidney and liver donors by sex. Data on donor sex was provided by 55 and 32 countries for kidney and liver donors, respectively.

Supplementary Table S3 Distribution of solid organ transplant recipients by sex. Data on recipient sex for kidney, liver, heart, lung, pancreas transplants was provided by 62, 56 countries, 47, 42 and 37 countries, respectively.

## Abbreviations

CD-P-TO, European Committee on Organ Transplantation of the Council of Europe; DBD, donor/donation after brain death; DD, deceased donor; DCDD, donor/donation after circulatory determination of death; LD, living donor; LKD, living kidney donation; LLD, living liver donation; ONT, Organización Nacional de Trasplantes; PMP, per million population.
