# Supplementary material for: An Analysis by the European Committee on Organ Transplantation of the Council of Europe Outlining the International Landscape of Donors and Recipients Sex in Solid Organ Transplantation
Source: Transpl Int. 2022 Jul 19;35:10322. doi: 10.3389/ti.2022.10322 (PMC9343585; doi:10.3389/ti.2022.10322)
Supplement: Supplementary file 1 [file Table1.DOCX]

| **Country** | **Population** | **Deceased Donors**  **(DD)** | | | | **Donor after brain death**  **(DBD)** | | | **Donor after circulatory death**  **(DCDD)** | | |
| --- | --- | --- | --- | --- | --- | --- | --- | --- | --- | --- | --- |
|  |  | **n** | **pmp** | **Female**  **n** | **Female**  **%** | **n** | **Female**  **n** | **Female**  **%** | **n** | **Female**  **n** | **Female**  **%** |
| Algeria | 42,70 | 1 | 0,0 | 0 | 0,0 | 1 | 0 | 0,0 | 0 | 0 |  |
| Argentina | 45,10 | 883 | 19,6 | 356 | 40,3 | 883 | 356 | 40,3 | 0 | 0 |  |
| Armenia | 2,90 | 0 | 0,0 | 0 |  | 0 | 0 |  | 0 | 0 |  |
| Australia | 25,10 | 548 | 21,8 | 208 | 38,0 | 376 | 152 | 40,4 | 172 | 56 | 32,6 |
| Austria | 8,80 | 206 | 23,4 | 97 | 47,1 | 185 | 88 | 47,6 | 21 | 9 | 42,9 |
| Belarus | 9,40 | 248 | 26,4 | 83 | 33,5 | 248 | 83 | 33,5 | 0 | 0 |  |
| Belgium | 11,60 | 321 | 27,7 | 125 | 38,9 | 211 | 80 | 37,9 | 110 | 45 | 40,9 |
| Brazil | 212,40 | 3767 | 17,7 | 1538 | 40,8 | 3767 | 1538 | 40,8 | 0 | 0 |  |
| Bulgaria | 7,00 | 16 | 2,3 | 6 | 37,5 | 16 | 6 | 37,5 | 0 | 0 |  |
| Chile | 18,30 | 188 | 10,3 | 70 | 37,2 | 188 | 70 | 37,2 | 0 | 0 |  |
| China | 1428,20 | 5818 | 4,1 | 1072 | 18,4 | 1906 | 340 | 17,8 | 3912 | 732 | 18,7 |
| Colombia | 49,80 | 417 | 8,4 | 157 | 37,6 | 417 | 157 | 37,6 | 0 | 0 |  |
| Costa Rica | 5,00 | 33 | 6,6 | 16 | 48,5 | 33 | 16 | 48,5 | 0 | 0 |  |
| Croatia | 4,10 | 131 | 32,0 | 54 | 41,2 | 131 | 54 | 41,2 | 0 | 0 |  |
| Cuba | 11,50 | 138 | 12,0 | 45 | 32,6 | 138 | 45 | 32,6 | 0 | 0 |  |
| Cyprus | 1,20 | 6 | 5,0 | 2 | 33,3 | 6 | 2 | 33,3 | 0 | 0 |  |
| Czech Republic | 10,60 | 288 | 27,2 | 114 | 39,6 | 270 | 105 | 38,9 | 18 | 9 | 50,0 |
| Denmark | 5,80 | 102 | 17,6 | 49 | 48,0 | 102 | 49 | 48,0 | 0 | 0 |  |
| Dominican Republic | 11,00 | 23 | 2,1 | 5 | 21,7 | 23 | 5 | 21,7 | 0 | 0 |  |
| Ecuador | 17,10 | 133 | 7,8 | 44 | 33,1 | 133 | 44 | 33,1 | 0 | 0 |  |
| Estonia | 1,30 | 25 | 19,2 | 9 | 36,0 | 25 | 9 | 36,0 | 0 | 0 |  |
| Finland | 5,60 | 145 | 25,9 | 65 | 44,8 | 145 | 65 | 44,8 | 0 | 0 |  |
| **Country** | **Population** | **Deceased Donors**  **(DD)** | | | | **Donor after brain death**  **(DBD)** | | | **Donor after circulatory death**  **(DCDD)** | | |
|  |  | **n** | **pmp** | **Female**  **n** | **Female**  **%** | **n** | **Female**  **n** | **Female**  **%** | **n** | **Female**  **n** | **Female**  **%** |
| France | 65,50 | 1924 | 29,4 | 813 | 42,3 | 1729 | 765 | 44,2 | 195 | 48 | 24,6 |
| Germany | 82,40 | 932 | 11,3 | 420 | 45,1 | 932 | 420 | 45,1 | 0 | 0 |  |
| Greece | 11,10 | 61 | 5,5 | 27 | 44,3 | 61 | 27 | 44,3 | 0 | 0 |  |
| Guatemala | 17,60 | 3 | 0,2 | 0 | 0,0 | 3 | 0 | 0,0 | 0 | 0 |  |
| Hungary | 9,70 | 180 | 18,6 | 71 | 39,4 | 180 | 71 | 39,4 | 0 | 0 |  |
| Iceland | 0,30 | 7 | 23,3 | 3 | 42,9 | 7 | 3 | 42,9 | 0 | 0 |  |
| India | 1368,70 | 715 | 0,5 | 189 | 26,4 | 715 | 189 | 26,4 | 0 | 0 |  |
| Ireland | 4,80 | 85 | 17,7 | 39 | 45,9 | 78 | 35 | 44,9 | 7 | 4 | 57,1 |
| Israel | 8,60 | 101 | 11,7 | 38 | 37,6 | 96 | 37 | 38,5 | 5 | 1 | 20,0 |
| Italy | 59,20 | 1495 | 25,3 | 626 | 41,9 | 1415 | 607 | 42,9 | 80 | 19 | 23,8 |
| Japan | 126,90 | 125 | 1,0 | 49 | 39,2 | 97 | 40 | 41,2 | 28 | 9 | 32,1 |
| Kuwait | 4,20 | 27 | 6,4 | 3 | 11,1 | 27 | 3 | 11,1 | 0 | 0 |  |
| Latvia | 1,90 | 19 | 10,0 | 10 | 52,6 | 19 | 10 | 52,6 | 0 | 0 |  |
| Lithuania | 2,90 | 52 | 17,9 | 19 | 36,5 | 52 | 19 | 36,5 | 0 | 0 |  |
| Luxembourg | 0,60 | 5 | 8,3 | 2 | 40,0 | 5 | 2 | 40,0 | 0 | 0 |  |
| Malaysia | 32,50 | 16 | 0,5 | 3 | 18,8 | 16 | 3 | 18,8 | 0 | 0 |  |
| Malta | 0,40 | 10 | 25,0 | 2 | 20,0 | 10 | 2 | 20,0 | 0 | 0 |  |
| Mexico | 132,30 | 500 | 3,8 | 196 | 39,2 | 500 | 196 | 39,2 | 0 | 0 |  |
| Mongolia | 3,20 | 9 | 2,8 | 2 | 22,2 | 9 | 2 | 22,2 | 0 | 0 |  |
| Netherlands | 17,10 | 258 | 15,1 | 121 | 46,9 | 106 | 59 | 55,7 | 152 | 62 | 40,8 |
| New Zealand | 4,80 | 74 | 15,4 | 31 | 41,9 | 61 | 28 | 45,9 | 13 | 3 | 23,1 |
| Nicaragua | 6,40 | 2 | 0,3 | 1 | 50,0 | 2 | 1 | 50,0 | 0 | 0 |  |
| Norway | 5,40 | 115 | 21,3 | 45 | 39,1 | 115 | 45 | 39,1 | 0 | 0 |  |
| Panama | 4,20 | 18 | 4,3 | 8 | 44,4 | 18 | 8 | 44,4 | 0 | 0 |  |
| Paraguay | 7,00 | 20 | 2,9 | 7 | 35,0 | 20 | 7 | 35,0 | 0 | 0 |  |
| Peru | 32,90 | 75 | 2,3 | 34 | 45,3 | 75 | 34 | 45,3 | 0 | 0 |  |
| Poland | 38,00 | 504 | 13,3 | 188 | 37,3 | 503 | 188 | 37,4 | 1 | 0 | 0,0 |
| **Country** | **Population** | **Deceased Donors**  **(DD)** | | | | **Donor after brain death**  **(DBD)** | | | **Donor after circulatory death**  **(DCDD)** | | |
|  |  | **n** | **pmp** | **Female**  **n** | **Female**  **%** | **n** | **Female**  **n** | **Female**  **%** | **n** | **Female**  **n** | **Female**  **%** |
| Portugal | 10,30 | 347 | 33,7 | 141 | 40,6 | 320 | 136 | 42,5 | 27 | 5 | 18,5 |
| Qatar | 2,70 | 8 | 3,0 | 0 | 0,0 | 8 | 0 | 0,0 | 0 | 0 |  |
| Republic of Moldova | 4,00 | 11 | 2,8 | 3 | 27,3 | 11 | 3 | 27,3 | 0 | 0 |  |
| Rep. North Macedonia | 2,10 | 3 | 1,4 |  |  | 3 |  |  | 0 | 0 |  |
| Romania | 19,50 | 85 | 4,4 | 28 | 32,9 | 85 | 28 | 32,9 | 0 | 0 |  |
| Russian Federation | 143,90 | 740 | 5,1 | 259 | 35,0 | 696 | 229 | 32,9 | 44 | 30 | 68,2 |
| Saudi Arabia | 34,10 | 114 | 3,3 | 15 | 13,2 | 114 | 15 | 13,2 | 0 | 0 |  |
| Slovakia | 5,50 | 98 | 17,8 | 34 | 34,7 | 98 | 34 | 34,7 | 0 | 0 |  |
| Slovenia | 2,10 | 44 | 21,0 | 24 | 54,5 | 44 | 24 | 54,5 | 0 | 0 |  |
| Spain | 46,40 | 2302 | 49,6 | 946 | 41,1 | 1557 | 711 | 45,7 | 745 | 235 | 31,5 |
| Sudan | 42,50 | 0 | 0,0 | 0 |  | 0 | 0 |  | 0 | 0 |  |
| Sweden | 10,10 | 198 | 19,6 | 75 | 37,9 | 194 | 75 | 38,7 | 4 | 0 | 0,0 |
| Switzerland | 8,60 | 157 | 18,3 | 61 | 38,9 | 100 | 44 | 44,0 | 57 | 17 | 29,8 |
| Syrian Arab Republic | 18,50 | 0 | 0,0 | 0 |  | 0 | 0 |  | 0 | 0 |  |
| Turkey | 83,00 | 499 | 6,0 | 182 | 36,5 | 499 | 182 | 36,5 | 0 | 0 |  |
| United Arab Emirates | 9,70 | 10 | 1,0 | 6 | 60,0 | 10 | 6 | 60,0 | 0 | 0 |  |
| United Kingdom | 67,00 | 1653 | 24,7 | 715 | 43,3 | 964 | 472 | 49,0 | 689 | 243 | 35,3 |
| US | 329,10 | 11870 | 36,1 | 4706 | 39,6 | 9152 | 3708 | 40,5 | 2718 | 998 | 36,7 |
| Uruguay | 3,50 | 75 | 21,4 | 32 | 42,7 | 75 | 32 | 42,7 | 0 | 0 |  |
| Venezuela | 32,80 | 0 | 0,0 | 0 |  | 0 | 0 |  | 0 | 0 |  |
