# Supplementary figures and images for: An Analysis by the European Committee on Organ Transplantation of the Council of Europe Outlining the International Landscape of Donors and Recipients Sex in Solid Organ Transplantation
Source: Transpl Int. 2022 Jul 19;35:10322. doi: 10.3389/ti.2022.10322 (PMC9343585; doi:10.3389/ti.2022.10322)

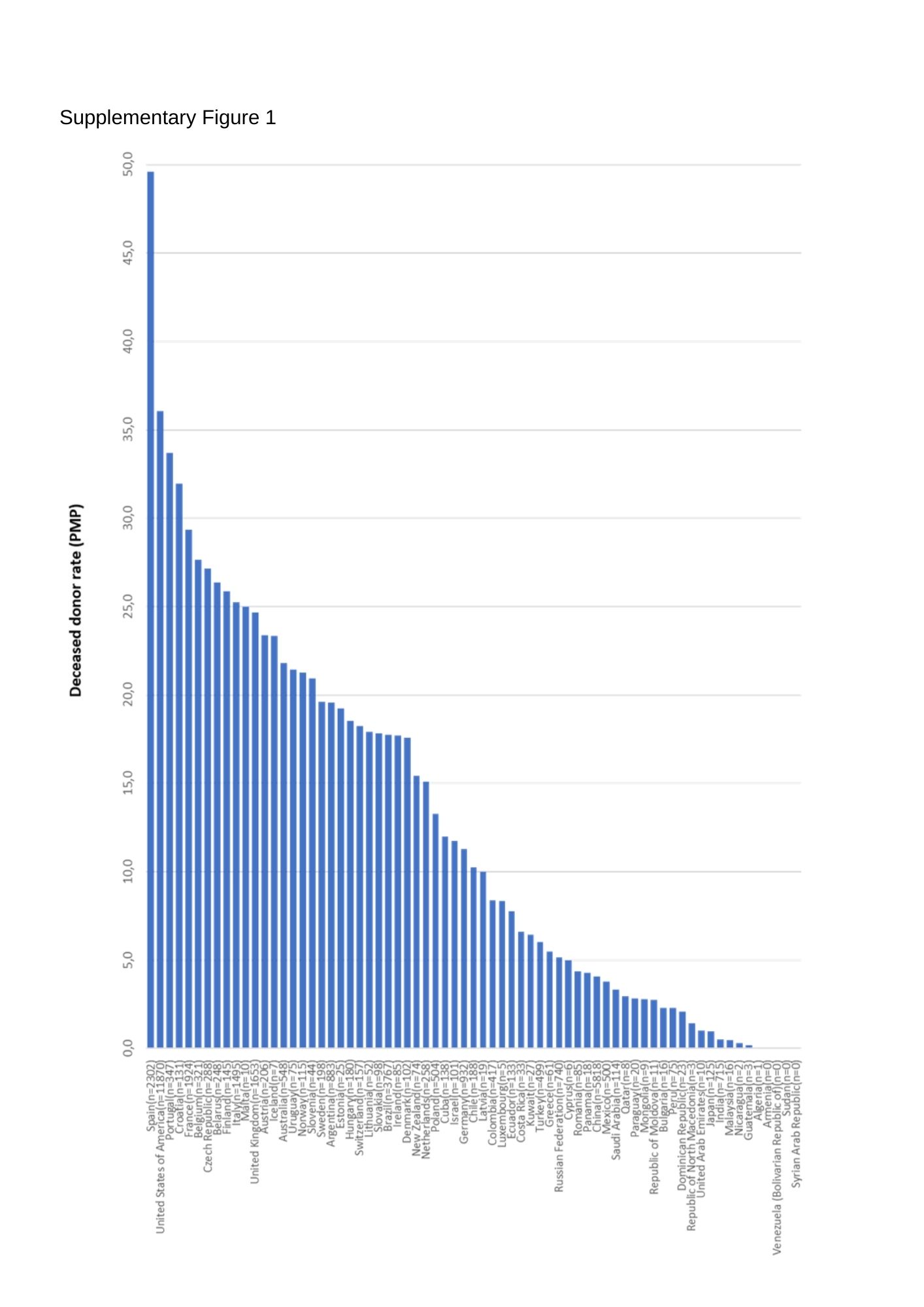

Supplement: Supplementary file 2 [file Image1.JPEG]
