# Supplementary material for: An Analysis by the European Committee on Organ Transplantation of the Council of Europe Outlining the International Landscape of Donors and Recipients Sex in Solid Organ Transplantation
Source: Transpl Int. 2022 Jul 19;35:10322. doi: 10.3389/ti.2022.10322 (PMC9343585; doi:10.3389/ti.2022.10322)
Supplement: Supplementary file 3 [file Table2.DOCX]

**KIDNEY LIVING DONORS**

| **Country** | **Population** | **Kidney living donors n** | **Kidney living donors pmp** | **Kidney living donors - Female n** | **Kidney living donors - Female %** |
| --- | --- | --- | --- | --- | --- |
| Algeria | 42,7 | 268 | 6,3 | 158 | 59,0 |
| Argentina | 45,1 | 351 | 7,8 | 150 | 42,7 |
| Armenia | 2,9 | 20 | 6,9 | 9 | 45,0 |
| Australia | 25,1 | 238 | 9,5 | 130 | 54,6 |
| Austria | 8,8 | 77 | 8,8 | 51 | 66,2 |
| Belarus | 9,4 | 6 | 0,6 | 4 | 66,7 |
| Belgium | 11,6 | 45 | 3,9 |  |  |
| Brazil | 212,4 | 1071 | 5,0 |  |  |
| Bulgaria | 7,0 | 13 | 1,9 | 9 | 69,2 |
| Chile | 18,3 | 99 | 5,4 | 56 | 56,6 |
| China | 1428,2 | 1735 | 1,2 | 1157 | 66,7 |
| Colombia | 49,8 | 179 | 3,6 |  |  |
| Costa Rica | 5,0 | 43 | 8,6 | 18 | 41,9 |
| Croatia | 4,1 | 7 | 1,7 |  |  |
| Cuba | 11,5 | 25 | 2,2 | 15 | 60,0 |
| Cyprus | 1,2 | 9 | 7,5 | 9 | 100,0 |
| Czech Republic | 10,6 | 49 | 4,6 | 29 | 59,2 |
| Denmark | 5,8 | 87 | 15,0 | 57 | 65,5 |
| Dominican Republic | 11,0 | 44 | 4,0 | 19 | 43,2 |
| Ecuador | 17,1 | 5 | 0,3 | 0 | 0,0 |
| Estonia | 1,3 | 2 | 1,5 | 2 | 100,0 |
| Finland | 5,6 | 25 | 4,5 | 13 | 52,0 |
| France | 65,5 | 510 | 7,8 | 309 | 60,6 |
| Germany | 82,4 | 520 | 6,3 |  |  |
| Greece | 11,1 | 68 | 6,1 | 49 | 72,1 |
| Guatemala | 17,6 | 103 | 5,9 |  |  |
| Hungary | 9,7 | 30 | 3,1 | 12 | 40,0 |
| Iceland | 0,3 | 8 | 26,7 | 5 | 62,5 |
| India | 1368,7 | 8613 | 6,3 | 5633 | 65,4 |
| Ireland | 4,8 | 25 | 5,2 | 16 | 64,0 |
| Israel | 8,6 | 248 | 28,8 | 91 | 36,7 |
| Italy | 59,2 | 340 | 5,7 | 115 | 33,8 |
| Japan | 126,9 | 1683 | 13,3 |  |  |
| Kuwait | 4,2 | 53 | 12,6 | 17 | 32,1 |
| Latvia | 1,9 | 6 | 3,2 | 3 | 50,0 |
| Lithuania | 2,9 | 7 | 2,4 | 2 | 28,6 |
| Luxembourg | 0,6 | 0 | 0,0 | 0 |  |
| Malaysia | 32,5 | 100 | 3,1 | 61 | 61,0 |
| Malta | 0,4 | 5 | 12,5 | 2 | 40,0 |
| Mexico | 132,3 | 2038 | 15,4 | 1075 | 52,7 |
| Mongolia | 3,2 | 18 | 5,6 | 6 | 33,3 |
| **Country** | **Population** | **Kidney living donors n** | **Kidney living donors pmp** | **Kidney living donors - Female n** | **Kidney living donors - Female %** |
| Netherlands | 17,1 | 501 | 29,3 |  |  |
| New Zealand | 4,8 | 91 | 19,0 | 53 | 58,2 |
| Nicaragua | 6,4 | 9 | 1,4 | 5 | 55,6 |
| Norway | 5,4 | 67 | 12,4 | 35 | 52,2 |
| Panama | 4,2 | 12 | 2,9 | 7 | 58,3 |
| Paraguay | 7,0 | 6 | 0,9 | 4 | 66,7 |
| Peru | 32,90 | 52 | 1,6 | 32 | 61,5 |
| Poland | 38,00 | 52 | 1,4 | 31 | 59,6 |
| Portugal | 10,30 | 75 | 7,3 | 54 | 72,0 |
| Qatar | 2,70 | 38 | 14,1 | 16 | 42,1 |
| Republic of Moldova | 4,00 | 3 | 0,8 | 2 | 66,7 |
| Rep. North Macedonia | 2,10 | 10 | 4,8 | 8 | 80,0 |
| Romania | 19,50 | 57 | 2,9 |  |  |
| Russian Federation | 143,90 | 183 | 1,3 | 139 | 76,0 |
| Saudi Arabia | 34,10 | 981 | 28,8 |  |  |
| Slovakia | 5,50 | 21 | 3,8 | 17 | 81,0 |
| Slovenia | 2,10 | 0 | 0,0 | 0 |  |
| Spain | 46,40 | 335 | 7,2 | 217 | 64,8 |
| Sudan | 42,50 | 313 | 7,4 | 164 | 52,4 |
| Sweden | 10,10 | 147 | 14,6 | 102 | 69,4 |
| Switzerland | 8,60 | 108 | 12,6 |  |  |
| Syrian Arab Republic | 18,50 | 275 | 14,9 |  |  |
| Turkey | 83,00 | 3055 | 36,8 | 1634 | 53,5 |
| United Arab Emirates | 9,70 | 48 | 4,9 | 27 | 56,3 |
| United Kingdom | 67,00 | 1022 | 15,3 | 538 | 52,6 |
| US | 329,10 | 6863 | 20,9 | 4473 | 65,2 |
| Uruguay | 3,50 | 13 | 3,7 | 8 | 61,5 |
| Venezuela | 32,80 | 6 | 0,2 | 2 | 33,3 |

**LIVER LIVING DONORS**

| **Country** | **Population** | **Liver living donors n** | **Liver living donors pmp** | **Liver living donors - Female n** | **Liver living donors - Female %** |
| --- | --- | --- | --- | --- | --- |
| Algeria | 42,70 | 11 | 0,3 | 4 | 36,4 |
| Argentina | 45,10 | 41 | 0,9 | 22 | 53,7 |
| Armenia | 2,90 | 3 | 1,0 | 2 | 66,7 |
| Australia | 25,10 | 1 | 0,0 | 1 | 100,0 |
| Austria | 8,80 | 12 | 1,4 | 7 | 58,3 |
| Belarus | 9,40 | 8 | 0,9 | 5 | 62,5 |
| Belgium | 11,60 | 27 | 2,3 |  |  |
| Brazil | 212,40 | 156 | 0,7 |  |  |
| Bulgaria | 7,00 | 3 | 0,4 | 2 | 66,7 |
| Chile | 18,30 | 19 | 1,0 | 5 | 26,3 |
| China | 1428,20 | 831 | 0,6 | 478 | 57,5 |
| Colombia | 49,80 | 55 | 1,1 |  |  |
| Costa Rica | 5,00 | 0 | 0,0 | 0 |  |
| Croatia | 4,10 | 0 | 0,0 | 0 |  |
| Cuba | 11,50 | 1 | 0,1 | 1 | 100,0 |
| Cyprus | 1,20 | 0 | 0,0 | 0 |  |
| Czech Republic | 10,60 | 0 | 0,0 | 0 |  |
| Denmark | 5,80 | 0 | 0,0 | 0 |  |
| Dominican Republic | 11,00 | 0 | 0,0 | 0 |  |
| Ecuador | 17,10 | 0 | 0,0 | 0 |  |
| Estonia | 1,30 | 0 | 0,0 | 0 |  |
| Finland | 5,60 | 0 | 0,0 | 0 |  |
| France | 65,50 | 4 | 0,1 | 2 | 50,0 |
| Germany | 82,40 | 54 | 0,7 |  |  |
| Greece | 11,10 | 0 | 0,0 | 0 |  |
| Guatemala | 17,60 | 0 | 0,0 | 0 |  |
| Hungary | 9,70 | 0 | 0,0 | 0 |  |
| Iceland | 0,30 | 0 | 0,0 | 0 |  |
| India | 1368,70 | 1993 | 1,5 | 1084 | 54,4 |
| Ireland | 4,80 | 0 | 0,0 | 0 |  |
| Israel | 8,60 | 12 | 1,4 | 4 | 33,3 |
| Italy | 59,20 | 24 | 0,4 | 11 | 45,8 |
| Japan | 126,90 | 307 | 2,4 | 158 | 51,5 |
| Kuwait | 4,20 | 0 | 0,0 | 0 |  |
| Latvia | 1,90 | 0 | 0,0 | 0 |  |
| Lithuania | 2,90 | 0 | 0,0 | 0 |  |
| Luxembourg | 0,60 | 0 | 0,0 | 0 |  |
| Malaysia | 32,50 | 5 | 0,2 | 3 | 60,0 |
| Malta | 0,40 | 0 | 0,0 | 0 |  |
| Mexico | 132,30 | 10 | 0,1 | 3 | 30,0 |
| Mongolia | 3,20 | 31 | 9,7 | 16 | 51,6 |
| **Country** | **Population** | **Liver living donors n** | **Liver living donors pmp** | **Liver living donors - Female n** | **Liver living donors - Female %** |
| Netherlands | 17,10 | 22 | 1,3 |  |  |
| New Zealand | 4,80 | 0 | 0,0 | 0 |  |
| Nicaragua | 6,40 | 0 | 0,0 | 0 |  |
| Norway | 5,40 | 0 | 0,0 | 0 |  |
| Panama | 4,20 | 0 | 0,0 | 0 |  |
| Paraguay | 7,00 | 0 | 0,0 | 0 |  |
| Peru | 32,90 | 6 | 0,2 | 2 | 33,3 |
| Poland | 38,00 | 21 | 0,6 | 16 | 76,2 |
| Portugal | 10,30 | 3 | 0,3 | 0 | 0,0 |
| Qatar | 2,70 | 1 | 0,4 | 0 | 0,0 |
| Republic of Moldova | 4,00 | 2 | 0,5 | 0 | 0,0 |
| Rep. North Macedonia | 2,10 | 0 | 0,0 | 0 |  |
| Romania | 19,50 | 8 | 0,4 |  |  |
| Russian Federation | 143,90 | 147 | 1,0 | 95 | 64,6 |
| Saudi Arabia | 34,10 | 241 | 7,1 |  |  |
| Slovakia | 5,50 | 0 | 0,0 | 0 |  |
| Slovenia | 2,10 | 0 | 0,0 | 0 |  |
| Spain | 46,40 | 21 | 0,5 | 7 | 33,3 |
| Sudan | 42,50 | 0 | 0,0 | 0 |  |
| Sweden | 10,10 | 3 | 0,3 | 2 | 66,7 |
| Switzerland | 8,60 | 1 | 0,1 |  |  |
| Syrian Arab Republic | 18,50 | 1 | 0,1 | 0 | 0,0 |
| Turkey | 83,00 | 1341 | 16,2 | 553 | 41,2 |
| United Arab Emirates | 9,70 | 8 | 0,8 | 4 | 50,0 |
| United Kingdom | 67,00 | 22 | 0,3 | 11 | 50,0 |
| US | 329,10 | 516 | 1,6 | 272 | 52,7 |
| Uruguay | 3,50 | 2 | 0,6 | 2 | 100,0 |
| Venezuela | 32,80 | 0 | 0,0 | 0 |  |
