# Supplementary material for: An Analysis by the European Committee on Organ Transplantation of the Council of Europe Outlining the International Landscape of Donors and Recipients Sex in Solid Organ Transplantation
Source: Transpl Int. 2022 Jul 19;35:10322. doi: 10.3389/ti.2022.10322 (PMC9343585; doi:10.3389/ti.2022.10322)
Supplement: Supplementary file 4 [file Table3.DOCX]

**KIDNEY RECIPIENTS**

| **Country** | **Population** | **Total number of patients transplanted** | **Kidney tx pmp** | **Female recipients n** | **Female recipients**  **%** |
| --- | --- | --- | --- | --- | --- |
| Algeria | 42,7 | 270 | 6,3 | 82 | 30,4 |
| Argentina | 45,1 | 1675 | 37,1 | 675 | 40,3 |
| Armenia | 2,9 | 20 | 6,9 | 8 | 40,0 |
| Australia | 25,1 | 1095 | 43,6 | 450 | 41,1 |
| Austria | 8,8 | 386 | 43,9 | 127 | 32,9 |
| Belarus | 9,4 | 376 | 40,0 | 160 | 42,6 |
| Belgium | 11,6 | 434 | 37,4 | 159 | 36,6 |
| Brazil | 212,4 | 6298 | 29,7 | 2420 | 38,4 |
| Bulgaria | 7,0 | 36 | 5,1 | 10 | 27,8 |
| Chile | 18,3 | 410 | 22,4 | 173 | 42,2 |
| China | 1428,2 | 12124 | 8,5 | 3563 | 29,4 |
| Colombia | 49,8 | 947 | 19,0 | 374 | 39,5 |
| Costa Rica | 5,0 | 84 | 16,8 |  |  |
| Croatia | 4,1 | 135 | 32,9 | 46 | 34,1 |
| Cuba | 11,5 | 195 | 17,0 |  |  |
| Cyprus | 1,2 | 15 | 12,5 | 4 | 26,7 |
| Czech Republic | 10,6 | 510 | 48,1 | 200 | 39,2 |
| Denmark | 5,8 | 276 | 47,6 | 110 | 39,9 |
| Dominican Republic | 11,0 | 86 | 7,8 | 29 | 33,7 |
| Ecuador | 17,1 | 234 | 13,7 | 98 | 41,9 |
| Estonia | 1,3 | 41 | 31,5 | 6 | 14,6 |
| Finland | 5,6 | 293 | 52,3 | 113 | 38,6 |
| France | 65,5 | 3643 | 55,6 | 1354 | 37,2 |
| Germany | 82,4 | 2132 | 25,9 | 780 | 36,6 |
| Greece | 11,1 | 178 | 16,0 | 63 | 35,4 |
| Guatemala | 17,6 | 109 | 6,2 |  |  |
| Hungary | 9,7 | 266 | 27,4 | 121 | 45,5 |
| Iceland | 0,3 | 10 | 33,3 | 2 | 20,0 |
| India | 1368,7 | 9751 | 7,1 | 2734 | 28,0 |
| Ireland | 4,8 | 153 | 31,9 | 50 | 32,7 |
| Israel | 8,6 | 411 | 47,8 | 118 | 28,7 |
| Italy | 59,2 | 2139 | 36,1 | 796 | 37,2 |
| Japan | 126,9 | 1913 | 15,1 |  |  |
| Kuwait | 4,2 | 103 | 24,5 | 35 | 34,0 |
| Latvia | 1,9 | 37 | 19,5 | 17 | 45,9 |
| Lithuania | 2,9 | 103 | 35,5 | 48 | 46,6 |
| Luxembourg | 0,6 | 0 | 0,0 | 0 |  |
| Malaysia | 32,5 | 130 | 4,0 | 62 | 47,7 |
| Malta | 0,4 | 16 | 40,0 | 6 | 37,5 |
| Mexico | 132,3 | 2976 | 22,5 | 1064 | 35,8 |
| **Country** | **Population** | **Total number of patients transplanted** | **Kidney tx pmp** | **Female recipients n** | **Female recipients**  **%** |
| Mongolia | 3,2 | 29 | 9,1 | 6 | 20,7 |
| Netherlands | 17,1 | 951 | 55,6 | 393 | 41,3 |
| New Zealand | 4,8 | 221 | 46,0 | 109 | 49,3 |
| Nicaragua | 6,4 | 11 | 1,7 | 4 | 36,4 |
| Norway | 5,4 | 258 | 47,8 | 79 | 30,6 |
| Panama | 4,2 | 31 | 7,4 | 13 | 41,9 |
| Paraguay | 7,0 | 40 | 5,7 | 11 | 27,5 |
| Peru | 32,9 | 189 | 5,7 | 77 | 40,7 |
| Poland | 38,0 | 983 | 25,9 | 351 | 35,7 |
| Portugal | 10,3 | 514 | 49,9 | 184 | 35,8 |
| Qatar | 2,7 | 48 | 17,8 | 18 | 37,5 |
| Republic of Moldova | 4,0 | 7 | 1,8 | 2 | 28,6 |
| Rep. North Macedonia | 2,1 | 16 | 7,6 | 4 | 25,0 |
| Romania | 19,5 | 211 | 10,8 |  |  |
| Russian Federation | 143,9 | 1473 | 10,2 | 686 | 46,6 |
| Saudi Arabia | 34,1 | 1118 | 32,8 | 430 | 38,5 |
| Slovakia | 5,5 | 197 | 35,8 | 71 | 36,0 |
| Slovenia | 2,1 | 38 | 18,1 | 8 | 21,1 |
| Spain | 46,4 | 3423 | 73,8 | 1179 | 34,4 |
| Sudan | 42,5 | 313 | 7,4 | 79 | 25,2 |
| Sweden | 10,1 | 476 | 47,1 | 152 | 31,9 |
| Switzerland | 8,6 | 340 | 39,5 | 130 | 38,2 |
| Syrian Arab Republic | 18,5 | 275 | 14,9 |  |  |
| Turkey | 83,0 | 3863 | 46,5 | 1369 | 35,4 |
| United Arab Emirates | 9,7 | 68 | 7,0 | 25 | 36,8 |
| United Kingdom | 67,0 | 3649 | 54,5 | 1413 | 38,7 |
| US | 329,1 | 24273 | 73,8 | 9460 | 39,0 |
| Uruguay | 3,5 | 155 | 44,3 | 65 | 41,9 |
| Venezuela | 32,8 | 6 | 0,2 | 2 | 33,3 |

**LIVER RECIPIENTS**

| **Country** | **Population** | **Total number of patients transplanted** | **Liver tx pmp** | **Female recipients n** | **Female recipients %** |
| --- | --- | --- | --- | --- | --- |
| Algeria | 42,70 | 12 | 0,3 | 5 | 41,7 |
| Argentina | 45,10 | 504 | 11,2 | 193 | 38,3 |
| Armenia | 2,90 | 3 | 1,0 | 2 | 66,7 |
| Australia | 25,10 | 310 | 12,4 | 97 | 31,3 |
| Austria | 8,80 | 151 | 17,2 | 33 | 21,9 |
| Belarus | 9,40 | 93 | 9,9 | 39 | 41,9 |
| Belgium | 11,60 | 289 | 24,9 | 113 | 39,1 |
| Brazil | 212,40 | 2265 | 10,7 | 762 | 33,6 |
| Bulgaria | 7,00 | 14 | 2,0 | 1 | 7,1 |
| Chile | 18,30 | 164 | 9,0 | 79 | 48,2 |
| China | 1428,20 | 6170 | 4,3 | 1544 | 25,0 |
| Colombia | 49,80 | 261 | 5,2 | 131 | 50,2 |
| Costa Rica | 5,00 | 19 | 3,8 |  |  |
| Croatia | 4,10 | 123 | 30,0 | 35 | 28,5 |
| Cuba | 11,50 | 10 | 0,9 |  |  |
| Cyprus | 1,20 | 0 | 0,0 | 0 |  |
| Czech Republic | 10,60 | 197 | 18,6 | 86 | 43,7 |
| Denmark | 5,80 | 64 | 11,0 | 24 | 37,5 |
| Dominican Republic | 11,00 | 5 | 0,5 | 3 | 60,0 |
| Ecuador | 17,10 | 27 | 1,6 | 11 | 40,7 |
| Estonia | 1,30 | 10 | 7,7 | 1 | 10,0 |
| Finland | 5,60 | 64 | 11,4 | 26 | 40,6 |
| France | 65,50 | 1356 | 20,7 | 404 | 29,8 |
| Germany | 82,40 | 831 | 10,1 | 311 | 37,4 |
| Greece | 11,10 | 33 | 3,0 | 5 | 15,2 |
| Guatemala | 17,60 | 0 | 0,0 | 0 |  |
| Hungary | 9,70 | 79 | 8,1 | 32 | 40,5 |
| Iceland | 0,30 | 0 | 0,0 | 0 |  |
| India | 1368,70 | 2592 | 1,9 | 665 | 25,7 |
| Ireland | 4,80 | 66 | 13,8 | 23 | 34,8 |
| Israel | 8,60 | 99 | 11,5 | 35 | 35,4 |
| Italy | 59,20 | 1301 | 22,0 | 356 | 27,4 |
| Japan | 126,90 | 395 | 3,1 | 212 | 53,7 |
| Kuwait | 4,20 | 6 | 1,4 | 2 | 33,3 |
| Latvia | 1,90 | 2 | 1,1 | 0 | 0,0 |
| Lithuania | 2,90 | 17 | 5,9 | 8 | 47,1 |
| Luxembourg | 0,60 | 0 | 0,0 | 0 |  |
| Malaysia | 32,50 | 8 | 0,2 | 3 | 37,5 |
| Malta | 0,40 | 0 | 0,0 | 0 |  |
| Mexico | 132,30 | 223 | 1,7 | 126 | 56,5 |
| **Country** | **Population** | **Total number of patients transplanted** | **Liver tx pmp** | **Female recipients n** | **Female recipients %** |
| Mongolia | 3,20 | 39 | 12,2 | 12 | 30,8 |
| Netherlands | 17,10 | 190 | 11,1 | 77 | 40,5 |
| New Zealand | 4,80 | 58 | 12,1 | 18 | 31,0 |
| Nicaragua | 6,40 | 0 | 0,0 | 0 |  |
| Norway | 5,40 | 94 | 17,4 | 37 | 39,4 |
| Panama | 4,20 | 9 | 2,1 | 5 | 55,6 |
| Paraguay | 7,00 | 2 | 0,3 | 1 | 50,0 |
| Peru | 32,90 | 53 | 1,6 | 19 | 35,8 |
| Poland | 38,00 | 352 | 9,3 | 149 | 42,3 |
| Portugal | 10,30 | 240 | 23,3 | 68 | 28,3 |
| Qatar | 2,70 | 8 | 3,0 | 0 | 0,0 |
| Republic of Moldova | 4,00 | 7 | 1,8 | 4 | 57,1 |
| Rep. North Macedonia | 2,10 | 0 | 0,0 | 0 |  |
| Romania | 19,50 | 75 | 3,8 |  |  |
| Russian Federation | 143,90 | 584 | 4,1 | 245 | 42,0 |
| Saudi Arabia | 34,10 | 319 | 9,4 |  |  |
| Slovakia | 5,50 | 41 | 7,5 | 16 | 39,0 |
| Slovenia | 2,10 | 24 | 11,4 | 11 | 45,8 |
| Spain | 46,40 | 1227 | 26,4 | 326 | 26,6 |
| Sudan | 42,50 | 0 | 0,0 | 0 |  |
| Sweden | 10,10 | 183 | 18,1 | 76 | 41,5 |
| Switzerland | 8,60 | 168 | 19,5 | 48 | 28,6 |
| Syrian Arab Republic | 18,50 | 1 | 0,1 | 0 | 0,0 |
| Turkey | 83,00 | 1776 | 21,4 | 596 | 33,6 |
| United Arab Emirates | 9,70 | 16 | 1,6 | 8 | 50,0 |
| United Kingdom | 67,00 | 971 | 14,5 | 395 | 40,7 |
| US | 329,10 | 8896 | 27,0 | 3300 | 37,1 |
| Uruguay | 3,50 | 23 | 6,6 | 6 | 26,1 |
| Venezuela | 32,80 | 0 | 0,0 | 0 |  |

**HEART RECIPIENTS**

| **Country** | **Population** | **Total number of patients transplanted** | **Heart tx pmp** | **Female recipients n** | **Female recipients %** |
| --- | --- | --- | --- | --- | --- |
| Algeria | 42,70 | 0 | 0,0 | 0 |  |
| Argentina | 45,10 | 124 | 2,7 | 42 | 33,9 |
| Armenia | 2,90 | 0 | 0,0 | 0 |  |
| Australia | 25,10 | 113 | 4,5 | 31 | 27,4 |
| Austria | 8,80 | 67 | 7,6 | 12 | 17,9 |
| Belarus | 9,40 | 52 | 5,5 | 13 | 25,0 |
| Belgium | 11,60 | 84 | 7,2 | 22 | 26,2 |
| Brazil | 212,40 | 383 | 1,8 | 109 | 28,5 |
| Bulgaria | 7,00 | 2 | 0,3 | 0 | 0,0 |
| Chile | 18,30 | 45 | 2,5 | 18 | 40,0 |
| China | 1428,20 | 679 | 0,5 | 172 | 25,3 |
| Colombia | 49,80 | 74 | 1,5 | 22 | 29,7 |
| Costa Rica | 5,00 | 1 | 0,2 |  |  |
| Croatia | 4,10 | 38 | 9,3 | 10 | 26,3 |
| Cuba | 11,50 | 1 | 0,1 | 0 | 0,0 |
| Cyprus | 1,20 | 0 | 0,0 | 0 |  |
| Czech Republic | 10,60 | 74 | 7,0 | 16 | 21,6 |
| Denmark | 5,80 | 30 | 5,2 | 10 | 33,3 |
| Dominican Republic | 11,00 | 0 | 0,0 | 0 |  |
| Ecuador | 17,10 | 0 | 0,0 | 0 |  |
| Estonia | 1,30 | 0 | 0,0 | 0 |  |
| Finland | 5,60 | 30 | 5,4 | 8 | 26,7 |
| France | 65,50 | 434 | 6,6 | 106 | 24,4 |
| Germany | 82,40 | 344 | 4,2 | 111 | 32,3 |
| Greece | 11,10 | 15 | 1,4 | 3 | 20,0 |
| Guatemala | 17,60 | 0 | 0,0 |  |  |
| Hungary | 9,70 | 72 | 7,4 | 21 | 29,2 |
| Iceland | 0,30 | 0 | 0,0 | 0 |  |
| India | 1368,70 | 187 | 0,1 | 47 | 25,1 |
| Ireland | 4,80 | 15 | 3,1 | 4 | 26,7 |
| Israel | 8,60 | 22 | 2,6 | 4 | 18,2 |
| Italy | 59,20 | 245 | 4,1 | 63 | 25,7 |
| Japan | 126,90 | 84 | 0,7 | 29 | 34,5 |
| Kuwait | 4,20 | 1 | 0,2 | 0 | 0,0 |
| Latvia | 1,90 | 0 | 0,0 | 0 |  |
| Lithuania | 2,90 | 8 | 2,8 | 0 | 0,0 |
| Luxembourg | 0,60 | 0 | 0,0 | 0 |  |
| Malaysia | 32,50 | 0 | 0,0 | 0 |  |
| Malta | 0,40 | 1 | 2,5 | 0 | 0,0 |
| Mexico | 132,30 | 33 | 0,2 | 9 | 27,3 |
| **Country** | **Population** | **Total number of patients transplanted** | **Heart tx pmp** | **Female recipients n** | **Female recipients %** |
| Mongolia | 3,20 | 0 | 0,0 | 0 |  |
| Netherlands | 17,10 | 38 | 2,2 | 11 | 28,9 |
| New Zealand | 4,80 | 17 | 3,5 | 4 | 23,5 |
| Nicaragua | 6,40 | 0 | 0,0 | 0 |  |
| Norway | 5,40 | 43 | 8,0 | 9 | 20,9 |
| Panama | 4,20 | 0 | 0,0 | 0 |  |
| Paraguay | 7,00 | 5 | 0,7 | 0 | 0,0 |
| Peru | 32,90 | 15 | 0,5 | 5 | 33,3 |
| Poland | 38,00 | 145 | 3,8 | 33 | 22,8 |
| Portugal | 10,30 | 29 | 2,8 | 10 | 34,5 |
| Qatar | 2,70 | 0 | 0,0 | 0 |  |
| Republic of Moldova | 4,00 | 0 | 0,0 | 0 |  |
| Rep. North Macedonia | 2,10 | 0 | 0,0 | 0 |  |
| Romania | 19,50 | 6 | 0,3 |  |  |
| Russian Federation | 143,90 | 337 | 2,3 | 54 | 16,0 |
| Saudi Arabia | 34,10 | 46 | 1,3 | 12 | 26,1 |
| Slovakia | 5,50 | 21 | 3,8 | 7 | 33,3 |
| Slovenia | 2,10 | 22 | 10,5 | 7 | 31,8 |
| Spain | 46,40 | 300 | 6,5 | 118 | 39,3 |
| Sudan | 42,50 | 0 | 0,0 | 0 |  |
| Sweden | 10,10 | 61 | 6,0 | 19 | 31,1 |
| Switzerland | 8,60 | 39 | 4,5 | 8 | 20,5 |
| Syrian Arab Republic | 18,50 | 0 | 0,0 | 0 |  |
| Turkey | 83,00 | 84 | 1,0 | 19 | 22,6 |
| United Arab Emirates | 9,70 | 5 | 0,5 | 1 | 20,0 |
| United Kingdom | 67,00 | 188 | 2,8 | 62 | 33,0 |
| US | 329,10 | 3597 | 10,9 | 1106 | 30,7 |
| Uruguay | 3,50 | 13 | 3,7 | 3 | 23,1 |
| Venezuela | 32,80 | 0 | 0,0 | 0 |  |

**LUNG RECIPIENTS**

| **Country** | **Population** | **Total number of patients transplanted** | **Lung tx pmp** | **Female recipients n** | **Female recipients %** |
| --- | --- | --- | --- | --- | --- |
| Algeria | 42,70 | 0 | 0,0 | 0 |  |
| Argentina | 45,10 | 36 | 0,8 | 17 | 47,2 |
| Armenia | 2,90 | 0 | 0,0 | 0 |  |
| Australia | 25,10 | 183 | 7,3 | 77 | 42,1 |
| Austria | 8,80 | 100 | 11,4 | 46 | 46,0 |
| Belarus | 9,40 | 9 | 1,0 | 3 | 33,3 |
| Belgium | 11,60 | 113 | 9,7 | 57 | 50,4 |
| Brazil | 212,40 | 106 | 0,5 | 52 | 49,1 |
| Bulgaria | 7,00 | 1 | 0,1 | 0 | 0,0 |
| Chile | 18,30 | 21 | 1,1 | 4 | 19,0 |
| China | 1428,20 | 489 | 0,3 | 87 | 17,8 |
| Colombia | 49,80 | 28 | 0,6 | 14 | 50,0 |
| Costa Rica | 5,00 | 2 | 0,4 |  |  |
| Croatia | 4,10 | 0 | 0,0 | 0 |  |
| Cuba | 11,50 | 0 | 0,0 | 0 |  |
| Cyprus | 1,20 | 0 | 0,0 | 0 |  |
| Czech Republic | 10,60 | 42 | 4,0 | 12 | 28,6 |
| Denmark | 5,80 | 30 | 5,2 | 17 | 56,7 |
| Dominican Republic | 11,00 | 0 | 0,0 | 0 |  |
| Ecuador | 17,10 | 1 | 0,1 | 1 | 100,0 |
| Estonia | 1,30 | 3 | 2,3 | 2 | 66,7 |
| Finland | 5,60 | 27 | 4,8 | 7 | 25,9 |
| France | 65,50 | 393 | 6,0 | 172 | 43,8 |
| Germany | 82,40 | 361 | 4,4 | 163 | 45,2 |
| Greece | 11,10 | 0 | 0,0 | 0 |  |
| Guatemala | 17,60 | 0 | 0,0 |  |  |
| Hungary | 9,70 | 18 | 1,9 | 8 | 44,4 |
| Iceland | 0,30 | 0 | 0,0 | 0 |  |
| India | 1368,70 | 114 | 0,1 | 52 | 45,6 |
| Ireland | 4,80 | 38 | 7,9 | 14 | 36,8 |
| Israel | 8,60 | 52 | 6,0 | 14 | 26,9 |
| Italy | 59,20 | 153 | 2,6 | 56 | 36,6 |
| Japan | 126,90 | 92 | 0,7 | 45 | 48,9 |
| Kuwait | 4,20 | 0 | 0,0 | 0 |  |
| Latvia | 1,90 | 0 | 0,0 | 0 |  |
| Lithuania | 2,90 | 1 | 0,3 | 1 | 100,0 |
| Luxembourg | 0,60 | 0 | 0,0 | 0 |  |
| Malaysia | 32,50 | 0 | 0,0 | 0 |  |
| Malta | 0,40 | 0 | 0,0 | 0 |  |
| Mexico | 132,30 | 7 | 0,1 | 3 | 42,9 |
| **Country** | **Population** | **Total number of patients transplanted** | **Lung tx pmp** | **Female recipients n** | **Female recipients %** |
| Mongolia | 3,20 | 0 | 0,0 | 0 |  |
| Netherlands | 17,10 | 105 | 6,1 | 52 | 49,5 |
| New Zealand | 4,80 | 26 | 5,4 | 14 | 53,8 |
| Nicaragua | 6,40 | 0 | 0,0 | 0 |  |
| Norway | 5,40 | 33 | 6,1 | 18 | 54,5 |
| Panama | 4,20 | 0 | 0,0 | 0 |  |
| Paraguay | 7,00 | 0 | 0,0 | 0 |  |
| Peru | 32,90 | 1 | 0,0 | 0 | 0,0 |
| Poland | 38,00 | 57 | 1,5 | 21 | 36,8 |
| Portugal | 10,30 | 39 | 3,8 | 13 | 33,3 |
| Qatar | 2,70 | 0 | 0,0 | 0 |  |
| Republic of Moldova | 4,00 | 0 | 0,0 | 0 |  |
| Rep. North Macedonia | 2,10 | 0 | 0,0 | 0 |  |
| Romania | 19,50 | 3 | 0,2 |  |  |
| Russian Federation | 143,90 | 25 | 0,2 | 7 | 28,0 |
| Saudi Arabia | 34,10 | 38 | 1,1 | 16 | 42,1 |
| Slovakia | 5,50 | 0 | 0,0 | 0 |  |
| Slovenia | 2,10 | 10 | 4,8 | 3 | 30,0 |
| Spain | 46,40 | 419 | 9,0 | 209 | 49,9 |
| Sudan | 42,50 | 0 | 0,0 | 0 |  |
| Sweden | 10,10 | 56 | 5,5 | 21 | 37,5 |
| Switzerland | 8,60 | 39 | 4,5 | 18 | 46,2 |
| Syrian Arab Republic | 18,50 | 0 | 0,0 | 0 |  |
| Turkey | 83,00 | 33 | 0,4 | 10 | 30,3 |
| United Arab Emirates | 9,70 | 4 | 0,4 | 3 | 75,0 |
| United Kingdom | 67,00 | 167 | 2,5 | 75 | 44,9 |
| US | 329,10 | 2759 | 8,4 | 1100 | 39,9 |
| Uruguay | 3,50 | 5 | 1,4 | 4 | 80,0 |
| Venezuela | 32,80 | 0 | 0,0 | 0 |  |

**PANCREAS RECIPIENTS**

| **Country** | **Population** | **Total number of patients transplanted** | **Pancreas tx pmp** | **Female recipients n** | **Female recipients %** |
| --- | --- | --- | --- | --- | --- |
| Algeria | 42,70 | 0 | 0,0 | 0 |  |
| Argentina | 45,10 | 74 | 1,6 | 33 | 44,6 |
| Armenia | 2,90 | 0 | 0,0 | 0 |  |
| Australia | 25,10 | 40 | 1,6 | 20 | 50,0 |
| Austria | 8,80 | 15 | 1,7 | 2 | 13,3 |
| Belarus | 9,40 | 1 | 0,1 | 1 | 100,0 |
| Belgium | 11,60 | 11 | 0,9 | 4 | 36,4 |
| Brazil | 212,40 | 177 | 0,8 | 75 | 42,4 |
| Bulgaria | 7,00 | 0 | 0,0 | 0 |  |
| Chile | 18,30 | 11 | 0,6 | 5 | 45,5 |
| China | 1428,20 | 0 | 0,0 | 0 |  |
| Colombia | 49,80 | 7 | 0,1 | 2 | 28,6 |
| Costa Rica | 5,00 | 0 | 0,0 | 0 |  |
| Croatia | 4,10 | 5 | 1,2 | 2 | 40,0 |
| Cuba | 11,50 | 0 | 0,0 | 0 |  |
| Cyprus | 1,20 | 0 | 0,0 | 0 |  |
| Czech Republic | 10,60 | 41 | 3,9 | 14 | 34,1 |
| Denmark | 5,80 | 6 | 1,0 | 4 | 66,7 |
| Dominican Republic | 11,00 | 0 | 0,0 | 0 |  |
| Ecuador | 17,10 | 0 | 0,0 | 0 |  |
| Estonia | 1,30 | 2 | 1,5 | 0 | 0,0 |
| Finland | 5,60 | 39 | 7,0 | 16 | 41,0 |
| France | 65,50 | 84 | 1,3 | 42 | 50,0 |
| Germany | 82,40 | 94 | 1,1 | 42 | 44,7 |
| Greece | 11,10 | 0 | 0,0 | 0 |  |
| Guatemala | 17,60 | 0 | 0,0 | 0 |  |
| Hungary | 9,70 | 5 | 0,5 | 2 | 40,0 |
| Iceland | 0,30 | 0 | 0,0 | 0 |  |
| India | 1368,70 | 22 | 0,0 | 6 | 27,3 |
| Ireland | 4,80 | 2 | 0,4 | 2 | 100,0 |
| Israel | 8,60 | 10 | 1,2 | 6 | 60,0 |
| Italy | 59,20 | 42 | 0,7 | 18 | 42,9 |
| Japan | 126,90 | 49 | 0,4 | 24 | 49,0 |
| Kuwait | 4,20 | 0 | 0,0 | 0 |  |
| Latvia | 1,90 | 0 | 0,0 | 0 |  |
| Lithuania | 2,90 | 0 | 0,0 | 0 |  |
| Luxembourg | 0,60 | 0 | 0,0 | 0 |  |
| Malaysia | 32,50 | 0 | 0,0 | 0 |  |
| Malta | 0,40 | 0 | 0,0 | 0 |  |
| Mexico | 132,30 | 2 | 0,0 | 2 | 100,0 |
| **Country** | **Population** | **Total number of patients transplanted** | **Pancreas tx pmp** | **Female recipients n** | **Female recipients %** |
| Mongolia | 3,20 | 0 | 0,0 | 0 |  |
| Netherlands | 17,10 | 25 | 1,5 | 12 | 48,0 |
| New Zealand | 4,80 | 4 | 0,8 | 3 | 75,0 |
| Nicaragua | 6,40 | 0 | 0,0 | 0 |  |
| Norway | 5,40 | 15 | 2,8 | 9 | 60,0 |
| Panama | 4,20 | 0 | 0,0 | 0 |  |
| Paraguay | 7,00 | 0 | 0,0 | 0 |  |
| Peru | 32,90 | 3 | 0,1 | 1 | 33,3 |
| Poland | 38,00 | 29 | 0,8 | 15 | 51,7 |
| Portugal | 10,30 | 25 | 2,4 | 9 | 36,0 |
| Qatar | 2,70 | 0 | 0,0 | 0 |  |
| Republic of Moldova | 4,00 | 0 | 0,0 | 0 |  |
| Rep. North Macedonia | 2,10 | 0 | 0,0 | 0 |  |
| Romania | 19,50 | 0 | 0,0 | 0 |  |
| Russian Federation | 143,90 | 10 | 0,1 | 5 | 50,0 |
| Saudi Arabia | 34,10 | 8 | 0,2 | 3 | 37,5 |
| Slovakia | 5,50 | 0 | 0,0 | 0 |  |
| Slovenia | 2,10 | 1 | 0,5 | 0 | 0,0 |
| Spain | 46,40 | 76 | 1,6 | 35 | 46,1 |
| Sudan | 42,50 | 0 | 0,0 | 0 |  |
| Sweden | 10,10 | 23 | 2,3 | 12 | 52,2 |
| Switzerland | 8,60 | 14 | 1,6 | 4 | 28,6 |
| Syrian Arab Republic | 18,50 | 0 | 0,0 | 0 |  |
| Turkey | 83,00 | 3 | 0,0 | 1 | 33,3 |
| United Arab Emirates | 9,70 | 0 | 0,0 | 0 |  |
| United Kingdom | 67,00 | 185 | 2,8 | 79 | 42,7 |
| US | 329,10 | 1015 | 3,1 | 397 | 39,1 |
| Uruguay | 3,50 | 0 | 0,0 | 0 |  |
| Venezuela | 32,80 | 0 | 0,0 | 0 |  |

**PATIENTS TRANSPLANTED**

| **Country** | **Population** | **Total number of patients transplanted** | **Patients tx pmp** | **Female recipients n** | **Female recipients %** |
| --- | --- | --- | --- | --- | --- |
| Algeria | 42,70 | 282 | 6,6 | 87 | 30,9 |
| Argentina | 45,10 | 2321 | 51,5 | 922 | 39,7 |
| Armenia | 2,90 | 23 | 7,9 | 10 | 43,5 |
| Australia | 25,10 | 1385 | 55,2 |  |  |
| Austria | 8,80 | 689 | 78,3 | 215 | 31,2 |
| Belarus | 9,40 | 530 | 56,4 | 215 | 40,6 |
| Belgium | 11,60 | 934 | 80,5 |  |  |
| Brazil | 212,40 | 9185 | 43,2 | 3407 | 37,1 |
| Bulgaria | 7,00 | 53 | 7,6 | 11 | 20,8 |
| Chile | 18,30 | 640 | 35,0 | 279 | 43,6 |
| China | 1428,20 | 19454 | 13,6 | 5362 | 27,6 |
| Colombia | 49,80 | 1303 | 26,2 | 536 | 41,1 |
| Costa Rica | 5,00 | 106 | 21,2 |  |  |
| Croatia | 4,10 | 296 | 72,2 |  |  |
| Cuba | 11,50 | 206 | 17,9 | 88 | 42,7 |
| Cyprus | 1,20 | 15 | 12,5 | 4 | 26,7 |
| Czech Republic | 10,60 | 818 | 77,2 | 181 | 22,1 |
| Denmark | 5,80 | 395 | 68,1 | 159 | 40,3 |
| Dominican Republic | 11,00 | 91 | 8,3 | 32 | 35,2 |
| Ecuador | 17,10 | 262 | 15,3 | 110 | 42,0 |
| Estonia | 1,30 | 54 | 41,5 | 9 | 16,7 |
| Finland | 5,60 | 413 | 73,8 | 156 | 37,8 |
| France | 65,50 | 5723 | 87,4 | 1998 | 34,9 |
| Germany | 82,40 | 3644 | 44,2 | 1351 | 37,1 |
| Greece | 11,10 | 226 | 20,4 | 71 | 31,4 |
| Guatemala | 17,60 | 109 | 6,2 |  |  |
| Hungary | 9,70 | 433 | 44,6 | 179 | 41,3 |
| Iceland | 0,30 | 10 | 33,3 | 2 | 20,0 |
| India | 1368,70 | 12625 | 9,2 | 3487 | 27,6 |
| Ireland | 4,80 | 296 | 61,7 |  |  |
| Israel | 8,60 | 575 | 66,9 | 189 | 32,9 |
| Italy | 59,20 | 3744 | 63,2 | 1240 | 33,1 |
| Japan | 126,90 | 2483 | 19,6 |  |  |
| Kuwait | 4,20 | 110 | 26,2 | 37 | 33,6 |
| Latvia | 1,90 | 39 | 20,5 | 17 | 43,6 |
| Lithuania | 2,90 | 129 | 44,5 | 57 | 44,2 |
| Luxembourg | 0,60 | 0 | 0,0 |  |  |
| Malaysia | 32,50 | 138 | 4,2 | 65 | 47,1 |
| Malta | 0,40 | 17 | 42,5 | 6 | 35,3 |
| Mexico | 132,30 | 3237 | 24,5 | 1204 | 37,2 |
| **Country** | **Population** | **Total number of patients transplanted** | **Patients tx pmp** | **Female recipients n** | **Female recipients %** |
| Mongolia | 3,20 | 68 | 21,3 | 18 | 26,5 |
| Netherlands | 17,10 | 1291 | 75,5 | 537 | 41,6 |
| New Zealand | 4,80 | 322 | 67,1 |  |  |
| Nicaragua | 6,40 | 11 | 1,7 | 4 | 36,4 |
| Norway | 5,40 | 436 | 80,7 | 149 | 34,2 |
| Panama | 4,20 | 40 | 9,5 | 18 | 45,0 |
| Paraguay | 7,00 | 47 | 6,7 | 12 | 25,5 |
| Peru | 32,90 | 258 | 7,8 | 101 | 39,1 |
| Poland | 38,00 | 1541 | 40,6 | 606 | 39,3 |
| Portugal | 10,30 | 820 | 79,6 | 272 | 33,2 |
| Qatar | 2,70 | 56 | 20,7 | 18 | 32,1 |
| Republic of Moldova | 4,00 | 14 | 3,5 | 6 | 42,9 |
| Rep. North Macedonia | 2,10 | 16 | 7,6 | 4 | 25,0 |
| Romania | 19,50 | 295 | 15,1 |  |  |
| Russian Federation | 143,90 | 2420 | 16,8 | 984 | 40,7 |
| Saudi Arabia | 34,10 | 1530 | 44,9 |  |  |
| Slovakia | 5,50 | 259 | 47,1 | 94 | 36,3 |
| Slovenia | 2,10 | 93 | 44,3 | 29 | 31,2 |
| Spain | 46,40 | 5326 | 114,8 | 1717 | 32,2 |
| Sudan | 42,50 | 313 | 7,4 | 79 | 25,2 |
| Sweden | 10,10 | 775 | 76,7 | 269 | 34,7 |
| Switzerland | 8,60 | 582 | 67,7 | 203 | 34,9 |
| Syrian Arab Republic | 18,50 | 275 | 14,9 |  |  |
| Turkey | 83,00 | 5729 | 69,0 | 1988 | 34,7 |
| United Arab Emirates | 9,70 | 93 | 9,6 | 37 | 39,8 |
| United Kingdom | 67,00 | 4992 | 74,5 | 1959 | 39,2 |
| US | 329,10 | 38433 | 116,8 | 14587 | 38,0 |
| Uruguay | 3,50 | 196 | 56,0 | 76 | 38,8 |
| Venezuela | 32,80 | 6 | 0,2 | 2 | 33,3 |
